# Retraction Note: Endocrine disruptors induce perturbations in endoplasmic reticulum and mitochondria of human pluripotent stem cell derivatives

**DOI:** 10.1038/s41467-019-08572-9

**Published:** 2019-02-02

**Authors:** Uthra Rajamani, Andrew R. Gross, Camille Ocampo, Allen M. Andres, Roberta A. Gottlieb, Dhruv Sareen

**Affiliations:** 1 Board of Governors—Regenerative Medicine Institute, Cedars-Sinai Medical Center, Los Angeles, CA 90048 USA; 2 Department of Biomedical Sciences, Cedars-Sinai Medical Center, Los Angeles, CA 90048 USA; 3 Department of Medicine, University of California, Los Angeles, CA 90048 USA; 4 Metabolism and Mitochondrial Research Core, Cedars-Sinai Medical Center, Los Angeles, CA 90048 USA; 5 Heart Institute, Cedars-Sinai Medical Center, Los Angeles, CA 90048 USA; 6 iPSC Core, The David Janet Polak Foundation Stem Cell Core Laboratory, Cedars-Sinai Medical Center, Los Angeles, CA 90048 USA

Retraction of: *Nature Communications*; 10.1038/s41467-017-00254-8; published online 09 August 2017

We the authors are retracting this Article as it has come to our attention that during figure assembly certain images were inappropriately processed in Figs. 3d, 4b and 6a of the Article. These data integrity issues undermine our full confidence in the integrity of the study and the authors therefore wish to retract the Article.

All the authors agree with the retraction.

